# Retaining Residual Ovarian Tissue following Ovarian Failure Has Limited Influence on Bone Loss in Aged Mice

**DOI:** 10.4061/2010/157323

**Published:** 2010-06-29

**Authors:** Zelieann R. Craig, Samuel L. Marion, Janet L. Funk, Mary L. Bouxsein, Patricia B. Hoyer

**Affiliations:** ^1^Department of Veterinary Biosciences, University of Illinois, 2001 S. Lincoln Avenue, Urbana, IL 61802, USA; ^2^Department of Physiology, University of Arizona, 1501 N Campbell Avenue, Tucson, AZ 85724, USA; ^3^Department of Medicine, University of Arizona, P.O. Box 245218, Tucson, AZ 85724, USA; ^4^Center for Advanced Orthopedic Studies, Beth Israel Deaconess Medical Center, Harvard Medical School, 330 Brookline Avenue, Boston, MA 02215, USA

## Abstract

Previous work showed that retaining residual ovarian tissue protects young mice from accelerated bone loss following ovarian failure. The present study was designed to determine whether this protection is also present in aged animals. Aged (9–12 months) C57BL/6Hsd female mice were divided into: CON (vehicle), VCD (160 mg/kg; 15d), or OVX (ovariectomized). Lumbar BMD was monitored by DXA and *μ*CT used to assess vertebral microarchitecture. BMD was not different between VCD and CON at any time point but was lower (*P* < .05) than baseline, starting 1 month after ovarian failure in VCD and OVX mice. Following *μ*CT analysis there were no differences between CON and VCD, but OVX mice had lower bone volume fraction, trabecular thickness, and a trend for decreased connectivity density. These findings provide evidence that retention of residual ovarian tissue may protect aged follicle-depleted mice from accelerated bone loss to a lesser extent than that observed in young mice.

## 1. Introduction

Life expectancy has increased such that today's women live on average a third of their lives in a postmenopausal stage. studying menopause has become of great importance due to its association with serious health risks such as osteoporosis [1], cardiovascular disease [2], ovarian cancer [3], and Alzheimer's disease [4]. Because women are expected to live a third of their lives after menopause, this translates to approximately three decades of increased risk for such disorders. 

In women, 17*β*-estradiol (E2) promotes osteoblast proliferation and collagen synthesis, blunts the effects of parathyroid hormone (PTH) to resorb calcium from bone, and directs 1,25 dihydroxy-vitamin D (1,25(OH)2D3) action to the intestines for calcium and phosphate absorption [5]. Accelerated loss of bone mineral density (BMD) has been associated with the loss of estrogen following menopause [6, 7]. Significant loss of bone mass leads to osteoporosis. Osteoporosis is a disease characterized by deterioration of bone tissue that leads to bone fragility and increased susceptibility to fractures [8, 9]. There are an estimated 10 million cases of osteoporosis in the United States, of which 80% are women. It is estimated that by the year 2025, annual costs associated with osteoporotic fractures will rise to $25.3 billion [10]. Therefore, studies aimed at understanding the biology of postmenopausal osteoporosis are of extreme importance.

The most common and widely accepted animal model for studying the effects of ovarian failure on bone physiology is the ovariectomized (OVX) rodent. However, one drawback to the OVX animal is that it cannot reproduce the effects of natural onset of menopause since removal of ovarian function is abrupt. The perimenopause may be associated with altered hormone levels and declines in bone mass [11]. Because the OVX animal cannot mimic that process, an animal model capable of reproducing both peri- and postmenopause could provide better information regarding postmenopausal osteoporosis.

An ovary-intact mouse model of menopause has been developed using the chemical 4-vinylcyclohexene diepoxide (VCD). VCD has been shown to selectively destroy the smallest preantral (primordial and primary) follicles in ovaries of mice and rats by accelerating the natural process of follicular atresia [12–15]. Because VCD does not target larger follicles, the animal continues to ovulate normally until no more follicles are available for recruitment. Thus, ovarian follicular depletion in the VCD-treated mouse (VCD mouse) is gradual. As with women undergoing perimenopause, VCD-treated mice show increased levels of FSH [16], declining levels of estrogen, and irregular estrous cycles [17] as they become follicle-depleted. Additionally, following ovarian failure residual ovarian tissue is retained. Therefore, the VCD-treated mouse is relevant for studies related to both perimenopausal and postmenopausal stages [18].

There has been an ongoing controversy as to whether residual ovarian tissue in postmenopausal women is steroidogenic [19–22]. This is important to resolve because, in the face of declining 17  *β*-estradiol, androgens produced by residual ovarian tissue could impact postmenopausal health in a negative or positive manner. Previous studies reported that dispersed cells collected from residual ovarian tissue in VCD-treated mice were capable of producing androstenedione *in vitro *[16] and that residual ovarian tissue expresses the enzymatic machinery necessary to synthesize androgens *de novo* [23]. Further, a previous paper has demonstrated that retaining residual ovarian tissue in young VCD-treated follicle-depleted mice protects them from accelerated bone loss following ovarian failure [24]. Therefore, the purpose of this study was to determine in aged follicle-depleted mice whether retaining residual ovarian tissue also influences bone loss relative to ovariectomized and normally cycling animals.

## 2. Materials and Methods

### 2.1. Animals

Cycling female C57BL/6Hsd mice (24 retired breeders; aged 9–12 months) were purchased from Harlan Sprague-Dawley, Inc. (Indianapolis, IN). Upon arrival, animals were housed in polycarbonate plastic cages, kept at 22 ± 2°C on 12L: 12D cycles and fed *ad libitum.* All animals were allowed to acclimate to the animal facilities for 1 week before the start of the experiment. Each animal was assigned randomly to one of three groups: (1) vehicle control, (2) VCD, or (3) ovariectomy. All experiments and methods were approved by the University of Arizona Institutional Animal Care and Use Committee (IACUC) and conformed to the Guide for the Care and Use of Experimental Animals.

### 2.2. Induction of Ovarian Failure (VCD Dosing)

Animals in the VCD group received VCD (160 mg/kg/d; i.p.; Sigma-Aldrich, St. Louis, MO, *n* = 8 mice) dissolved in sesame oil (vehicle) to induce ovarian failure. Animals in the control group (CON) received sesame oil only (i.p; Sigma-Aldrich; *n* = 8 mice). Mice were weighed and dosed daily for 15 days and estrous cyclicity was monitored by daily vaginal cytology in both groups. Ovarian failure was assigned when mice showed ≥10 days of persistent diestrus [17]. On-going estrous cyclicity was confirmed in the vehicle-treated controls (CON), and only VCD-treated mice underwent ovarian failure.

### 2.3. Surgical Removal of Ovaries (Ovariectomy)

To match the age at the onset ovarian failure and therefore the duration of exposure to estrogen deficiency, ovariectomy was performed in a separate group of untreated mice at the average time when mice in the VCD group experienced ovarian failure (day 35 after the onset of dosing in CON and VCD mice). Animals (*n* = 8) were placed under Ketamine/Xylazine anesthesia (“Ketaject”, Phoenix Pharmaceutical, Inc, St. Joseph, MO, USA; “AnaSed” from Lloyd Pharmaceuticals, Inc., Shenandoah, IA, USA), and the right and left flanks were shaved, and skin was cleaned with Povidone-iodine and 70% ethanol. Incisions were made through the skin, fascia, and abdominal wall, and the ovaries were identified and externalized. A ligature was placed at the ovary/oviduct boundary before each ovary was removed. The muscle wall and skin were closed using 6-0 and 3-0 braided silk suture, respectively. Tissumend II surgical glue (Veterinary Products Laboratories, Phoenix, AZ, USA) was applied over the incision to prevent reopening of the wound.

### 2.4. Tissue Collection

Animals were euthanized by CO_2_ inhalation followed by cervical dislocation on day 181 after the beginning of experiment. Skeletons were fixed in 10% neutral buffered formalin for 48 h and transferred to 70% ethanol for 24 h.

### 2.5. Bone Mineral Density (BMD) Measurements

Lumbar BMD was determined in vivo by Dual-energy X-ray Absorptiometry (DXA) using a PIXImus absorptiometer (GE Lunar, Madison, WI). After performing quality control measurements, animals were placed under anesthesia (Ketamine/Xylazine mix) and scanned. For each DXA scan, the region of interest (ROI) was manually set to include lumbar vertebrae 2–4.

### 2.6. Micrcomputed Tomography (*μ*CT)

Vertebrae were evaluated using a desktop *μ*CT imaging system (*μ*CT40; Scanco Medical AG, Bassersdorf, Switzerland) equipped with a 10-mm focal spot microfocus X-ray tube and images acquired with a 12 *μ*m isotropic voxel size, as previously described [25]. Morphometric variables describing bone microstructure were computed using direct 3D methods, including bone volume fraction (BV/TV, %), trabecular number (Tb.N, mm^−1^), trabecular thickness (Tb.Th, *μ*m), trabecular separation (Tb.Sp, *μ*m), connectivity density (ConnD, mm^−3^), and the structure model index (SMI), a measure of the plate- versus-rod-like nature of the trabecular structure.

### 2.7. Statistical Analysis

Data were compared using ANOVA followed by a Fischer's PLSD (Protected Least Significant Difference) post hoc test. Statistical tests were conducted using StatView for Windows (version 5.0, SAS Institute, Inc., Cary, NC) with the significance level set at *P* < .05 for all tests. An observation was considered a trend with *P* ≤ .09 but >.05.

## 3. Results

### 3.1. VCD-Induced Ovarian Failure

Retired breeders (9–12 months old) were dosed daily with VCD to induce premature ovarian failure. The average day of ovarian failure was d34.8 ± 1.3 (range: d31–39) after the onset of dosing. Thus, the ovaries from OVX mice were removed on d35 after the onset of dosing to match ovarian failure in VCD-treated mice. Estrous cyclicity was determined in vehicle control-treated animals to confirm ovarian cyclicity. Throughout the month preceding the end of the study (d151 − d181), cycling controls had estrous cycles of 5.08 ± 0.34 days (range: 3–7 days; estrus to estrus).

### 3.2. Changes in Bone Mineral Density in Mice following Ovarian Failure

**Figure 1:**
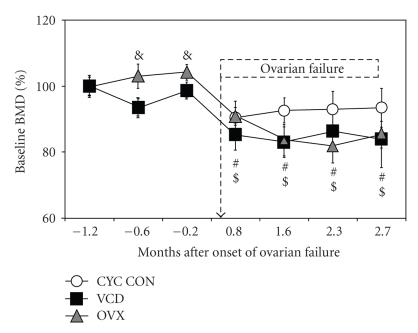
Temporal changes in lumbar BMD in aged mice. Aged C57BL/6Hsd female mice (9–12 months old) were dosed daily for 15 days with sesame oil (vehicle; CON) or VCD (160 mg/kg). An additional group of age-matched animals was not dosed but rather underwent bilateral ovariectomy (OVX) on d35 after the onset of dosing (matching ovarian failure in VCD-treated mice). BMD was determined using DXA as described in Section 2. Data are presented as percentage from baseline BMD ± SEM (*n* = 5–8). (#) and ($) indicate *P* < .05 from baseline BMD for VCD and OVX groups, respectively, and (&) indicates *P* < .05 versus OVX. The dashed arrow indicates onset of ovarian failure in VCD-treated mice, and the dashed box indicates BMD values measured after ovarian failure had occurred.

Lumbar bone mineral density (BMD) of aged VCD-treated and ovariectomized (OVX) mice was monitored by DXA and compared to that in age-matched cycling controls (Figure 1). Lumbar BMD was not different between VCD-treated animals and age-matched cycling controls at any of the measured time points. However, relative to cycling and VCD-treated animals, OVX had higher (*P* < .05) lumbar BMD at the two time points preceding ovarian failure, but was not different from the other groups at any other time point. In order to obtain information regarding the amount of bone density gain or loss within each group, all data was compared to their respective baseline BMD (Figure 1; CON: 0.051 ± 0.002 g/cm^3^; VCD: 0.051 ± 0.001 g/cm^3^; OVX: 0.052 ± 0.001 g/cm^3^). As expected for animals without ovarian failure, lumbar BMD in cycling control mice did not change (*P* > .05) over time until the end of the study (range: 0.051 ± 0.002 to 0.048 ± 0.003 g/cm^3^). However, both VCD-treated and OVX animals experienced a decline (*P* < .05) from baseline in BMD starting on d59 (age: ~11–14 months; ~1 month after ovarian failure or surgery in VCD and OVX mice) and continuing to the end of the study (VCD: 0.051 ± 0.001 to 0.043 ± 0.004 g/cm^3^, 16% loss; OVX: 0.052 ± 0.001 to 0.044 ± 0.002 g/cm^3^, 14.7% loss).

### 3.3. Evaluation of Trabecular Bone Microarchitecture in Aged Mice

**Figure 2:**
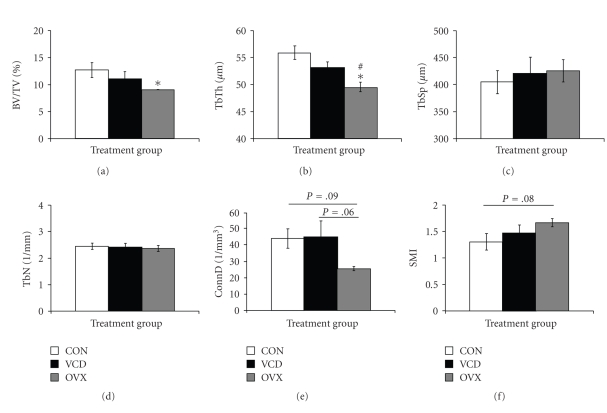
Trabecular microarchitecture in aged VCD-treated and ovariectomized mice. Aged C57BL/6Hsd female mice (9–12 months old) were dosed daily for 15 days with sesame oil (vehicle; CON) or VCD (160 mg/kg) or were ovariectomized on d35 after the start of the experiment. Trabecular bone microarchitecture of the fifth vertebral body was assessed by *μ*CT on samples collected at 5-month postovarian failure (age: 18.8 months; d181) as described in Section 2. Data are represented as mean ± SEM (*n* = 4–6). Significance was set at *P* < .05. The following variables were measured: bone volume fraction (BV/TV%), trabecular thickness (TbTh, *μ*m), trabecular separation (TbSp, *μ*m), trabecular number (TbN, 1/mm), connectivity density (ConnD, 1/mm^3^), and structure model index (SMI). (∗) indicates *P* < .05 versus CON; (#) indicates *P* < .05 versus VCD. Trends are represented by bars indicating the corresponding *P*-value.

**Figure 3:**
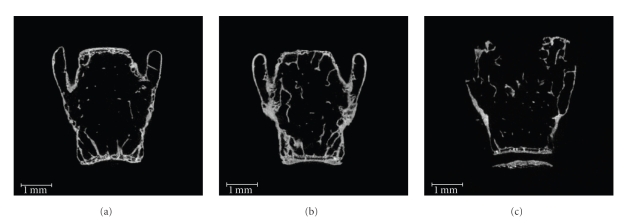
Representative two-dimensional frontal planar *μ*CT images showing changes in bone integrity of the fifth vertebral body of VCD-treated and ovariectomized mice. Aged C57BL/6Hsd female mice (9–12 months old) were dosed daily for 15 days with sesame oil (vehicle; CON) or VCD (160 mg/kg) or ovariectomized on d35 after the start of the experiment. Trabecular bone microarchitecture of the fifth vertebral body was assessed by *μ*CT on samples collected at 5-month postovarian failure (age: 18.8 months; d181) as described in Section 2. Shown are representative images of vertebral bodies excised from: (a) a cycling control-treated, (b) a VCD-treated, and (c) an ovariectomized animal. The scale bar represents 1.0 mm in all images.

Trabecular bone microarchitecture of the fifth lumbar vertebral body was assessed by *μ*CT after sacrifice on d181, after the onset of dosing (age: 18.8 months; Figure 2). There were no differences in any of the variables measured between cycling controls and VCD-treated mice (*P* > .05, Figure 2). However, OVX mice had lower (*P* < .05) BV/TV% and TbTh and trends for decreased ConnD (*P* = .09) and increased SMI when compared to the cycling control group (Figure 2). In addition, OVX mice had lower (*P* < .05) TbTh and a trend for decreased ConnD (*P* = .06) compared to VCD-treated mice. Two-dimensional frontal planar images of the vertebral bodies of ovariectomized animals showed obvious loss of integrity when compared to images from vertebrae of cycling control-treated and VCD-treated animals (Figure 3).

## 4. Discussion

This study was designed to determine whether retaining residual ovarian tissue influences the rate or extent of ovarian failure-induced bone loss in aged, follicle-depleted mice. In order to investigate the effect of ovarian failure on bone integrity, lumbar BMD of aged VCD-treated and ovariectomized mice was evaluated by DXA and *μ*CT and compared to that in age-matched cycling animals. There were no differences in lumbar BMD between VCD-treated and cycling control animals throughout the study. Lumbar BMD of cycling controls did not differ over time until the end of the study (age: 17.1 months). In comparison, within 1 month from ovarian failure, both VCD-treated and OVX mice experienced a decline in bone mineral density that remained stable until the end of the study. Trabecular bone microarchitecture, assessed by *μ*CT did not differ between aged VCD-treated mice and age-matched cycling animals. However, OVX animals showed deteriorated trabecular architecture relative to both age-matched cycling controls and VCD-treated mice. In particular, OVX mice had decreased bone volume fraction and trabecular thickness, along with trends for decreased connectivity density and a more rod-like architecture. Compared to trabecular microarchitecture values reported in a previous study [24], the 18.8-month-old cycling controls in the present study had 51.4% lower bone volume, 45.1% lower trabecular number, 84.8% greater trabecular separation, and 74.4% lower connectivity density than the 3-month-old mice of the same strain. Therefore, the lack of differences in trabecular microarchitecture may be explained by age-dependent loss of bone integrity in the cycling control group. 

 A previous study conducted in young mice supports the hypothesis that retaining residual ovarian tissue can afford protection against accelerated bone loss following ovarian failure [24]. In that study, young OVX mice showed reduced lumbar BMD when compared to cycling animals 1 month after ovariectomy while BMD from VCD-treated, follicle-depleted animals did not differ until approximately 3 months after VCD-induced ovarian failure. Results from that study also showed that despite observing significant trabecular deterioration in both mouse models of ovarian failure, changes in the OVX group were more dramatic than those observed in VCD-treated mice. One proposed mechanism for this protection is the production of androgens by residual ovarian tissue. Androgens derived from residual steroidogenesis may act directly on androgen receptors previously identified in bone tissue [26] or be converted to estrogens by aromatase expressed in bone [27–30] before acting on bone cells to protect against accelerated bone loss. Increased production of androstenedione has been shown in young VCD-treated, follicle-depleted mice [16, 23]. Also, young VCD-treated mice have been shown to have levels of androstenedione that are similar to those in cycling mice, but higher than those observed in age-matched OVX mice [24]. However, the present DXA data using aged animals suggests that this benefit may decrease with advancing age. Previous work showed that circulating androstenedione declines with age in cycling vehicle-treated mice [23]. This decline, although not completely understood, could be due to a decline in residual ovarian function as it has also been observed in aging cycling women [31] as well as in older postmenopausal women [32, 33]. A decline in residual ovarian function and circulating androstenedione may explain the diminished protection against bone loss observed in aged, follicle-depleted mice in the present experiment compared to that previously observed in young, follicle-depleted mice.

## 5. Conclusion

Previous studies have demonstrated that the presence of steroidogenically active residual ovarian tissue (versus removal by OVX) seems to play a beneficial role by retarding bone loss following ovarian failure in young mice [24]. Surprisingly, this protection was not as obvious in the present study, in which aged VCD and OVX animals had similar progression to significant bone loss when BMD was monitored by DXA. No differences in trabecular bone architecture were identified between aged VCD-treated and cycling control mice, suggesting that cycling controls had already undergone age-dependent loss of bone integrity at the time of measurement (age: 18.8 months). However, there were significant detrimental effects of ovarian failure on trabecular bone of OVX animals compared to cycling and VCD-treated mice. Therefore, these observations suggest that retaining residual ovarian tissue is less protective in aged follicle-depleted mice than in young. These findings further support the VCD-treated mouse as a useful model for studies related to menopause and osteoporosis.
